# Identification of deleterious and regulatory genomic variations in known asthma loci

**DOI:** 10.1186/s12931-018-0953-2

**Published:** 2018-12-12

**Authors:** Matthew D. C. Neville, Jihoon Choi, Jonathan Lieberman, Qing Ling Duan

**Affiliations:** 1 Department of Biomedical and Molecular Sciences, Queen’s University, Botterell Hall, Room 530 - 18 Stuart St, Kingston, ON K7L3N6 Canada; 2 School of Computing, Queen’s University, 557 Goodwin Hall, Room 531, Kingston, ON K7L 2N8 Canada

**Keywords:** Asthma, Heritability, Linkage disequilibrium, Causal variants, SNPs, eQTLs

## Abstract

**Background:**

Candidate gene and genome-wide association studies have identified hundreds of asthma risk loci. The majority of associated variants, however, are not known to have any biological function and are believed to represent markers rather than true causative mutations. We hypothesized that many of these associated markers are in linkage disequilibrium (LD) with the elusive causative variants.

**Methods:**

We compiled a comprehensive list of 449 asthma-associated variants previously reported in candidate gene and genome-wide association studies. Next, we identified all sequence variants located within the 305 unique genes using whole-genome sequencing data from the 1000 Genomes Project. Then, we calculated the LD between known asthma variants and the sequence variants within each gene. LD variants identified were then annotated to determine those that are potentially deleterious and/or functional (i.e. coding or regulatory effects on the encoded transcript or protein).

**Results:**

We identified 10,130 variants in LD (r^2^ > 0.6) with known asthma variants. Annotations of these LD variants revealed that several have potentially deleterious effects including frameshift, alternate splice site, stop-lost, and missense. Moreover, 24 of the LD variants have been reported to regulate gene expression as expression quantitative trait loci (eQTLs).

**Conclusions:**

This study is proof of concept that many of the genetic loci previously associated with complex diseases such as asthma are not causative but represent markers of disease, which are in LD with the elusive causative variants. We hereby report a number of potentially deleterious and regulatory variants that are in LD with the reported asthma loci. These reported LD variants could account for the original association signals with asthma and represent the true causative mutations at these loci.

**Electronic supplementary material:**

The online version of this article (10.1186/s12931-018-0953-2) contains supplementary material, which is available to authorized users.

## Background

Asthma is a chronic respiratory disease characterized by hyper-responsiveness of the bronchial muscles, inflammation, and reversible narrowing of the airways. It affects over 330 million individuals worldwide and is expected to increase to approximately 400 million by 2025 [1, 2]. Asthma is known to be multifactorial and polygenic in nature with numerous genetic and environmental risk factors. Twin studies have estimated that genetic factors contribute approximately 55–95% of asthma risk, known as its heritability [3–5]. To date, hundreds of genetic variants have been correlated with asthma and asthma related traits such as lung function and bronchial hyper-responsiveness. However, only a small portion of asthma risk is explained by known genetic loci. For example, in a recent study of 31 genetic variants previously associated for asthma across multiple populations, Vicente et al. determined that these collectively account for only approximately 2.6% of asthma heritability in the UK Biobank [6]. Thus, our understanding of the genetic determinants of asthma remains limited and further investigations to identify the missing heritability are necessary to facilitate better prevention, diagnosis and care of this chronic disease.

Several factors may account of the missing heritability of asthma such as epistasis, gene-environment interactions, rare variants, and epigenomics (i.e. non-sequence variations in the genome) [7]. In addition, the genetic variants previously correlated with asthma may not represent the true causative mutation. Instead, it is widely accepted that the majority of loci identified from genome-wide association studies (GWAS) as well as candidate gene studies are not causal but are genetic markers that tag causal variants [8]. This is supported by the fact that the majority of variants associated with complex diseases such as asthma have no known impact on the resulting transcripts or proteins, with over 80% from GWAS falling outside of protein coding regions [9]. Discovery of the causal variants underlying a disease would reveal the true genetic effect sizes [8] and help to facilitate the development of more accurate clinical tests for diagnosis and treatment of asthma [10].

In this study, we hypothesize that the causal mutations at numerous asthma-associated loci are likely in linkage disequilibrium (LD) with the associated markers, which refers to the non-random association of alleles at different loci [11]. We test this through the analysis of existing whole-genome sequencing data from large populations to identify genetic variants that are more likely to be causal and explain for the missing heritability of asthma. First, we compiled a comprehensive list of asthma-associated variants from earlier GWAS and candidate gene studies. Next, we identified all variants within these asthma loci using sequence data from the 1000 Genomes Project (1000GP) [12] and calculated LD between the asthma variants and the sequence variants. Then, both asthma variants and the LD variants were annotated for their predicted effects on the resulting transcript or protein. We hereby report a list of potentially deleterious and regulatory variants within known asthma loci that are in LD with and could account for the reported association signals. These results may improve our understanding of the underlying mechanisms of asthma, which will ultimately lead to better prevention and more efficient therapies.

## Methods

### Selection of asthma loci

Previously associated SNPs from asthma GWAS were obtained from the National Human Genome Research Institute-European Bioinformatics Institute (NHGRI-EBI) GWAS Catalog (October 10th, 2017 release) [13]. This manually curated GWAS catalog lists SNP-trait associations with *p*-values < 1.0 × 10^− 5^ in studies assaying at least 100,000 SNPs. We selected all SNPs linked to asthma and asthma severity traits: ‘Asthma’, ‘Adult asthma’, ‘Asthma (childhood onset)’, ‘Asthma (sex interaction)’, ‘Asthma and hay fever’, ‘Asthma or chronic obstructive pulmonary disease’, ‘Bronchial hyperresponsiveness in asthma’, and ‘Pulmonary function in asthmatics’.

A compilation of candidate genes were also selected from four previously published lists of asthma candidate gene studies [14–17]. A total of 148 genes that did not overlap with the asthma GWAS loci above were selected. We then proceeded to search for these candidate genes in PubMed using keywords including “asthma”, “polymorphism” or “snp”, and the gene name. All identified English language candidate gene studies reporting a genetic association for asthma or asthma severity published up to June 2016 were examined. Asthma-associated SNPs reported to a gene on our candidate gene list that were significant at the level defined by the authors of the study were included for further analysis.

### Genomic coordinates and 1000 genomes project

Starting with a comprehensive list of SNPs previously associated with asthma and related phenotypes from GWAS and candidate gene studies, we removed pseudogenes and uncharacterized loci unknown to the RefSeqGene database [18]. Next, the University of Santa Cruz (UCSC) Genome Browser [19] was used to identify the genomic coordinates of each gene using GRCh37/hg19 assembly of the human reference genome. The coordinates of each gene were extended by 5000 bp both 5′ and 3′ of the transcription start sites (TSS) and transcription end sites (TES) of each gene to include potential regulatory regions. These genomic coordinates were then applied to extract a complete list of sequence variations from the 1000GP Phase 3 whole genome sequencing data [12].

### Linkage disequilibrium analysis

At each locus, we calculated the LD between the asthma-associated SNP (reported by GWAS and candidate gene studies) and the newly identified variants from the 1000GP using Plink, version 1.9 [20]. A distance window of 1 Mb was used to determine LD, which excludes variants greater than 1 Mb apart. The r^2^ threshold for LD was set to ≥0.6.

### Functional annotation

The genomic variant annotations and functional effect prediction toolbox known as SnpEff (version 4.3q) [21] was used with the GRCh37.75 assembly to predict the effects of sequence variants identified from the 1000GP. To validate these annotations, we also used Ensembl’s Variant Effect Predictor (VEP), release 90, [22] and only concordant annotations between the two prediction tools were reported. For those variants with multiple annotations from each annotation tool (e.g. variants in regions impacted by multiple transcripts), only the most severe effect as ranked by SnpEff was selected. VEP was also used to access the pathogenicity predictions of missense mutations from the scoring tools Sorting Intolerant From Tolerant (SIFT) [23] and Polyphen-2 [24]. The Database of Genomic Structural Variations (dbVar) [25] was used to supplement annotations for structural variants.

RegulomeDB (version 1.1) [26] was used to evaluate the potential of variants to impact gene expression. RegulomeDB scores variants for their predicted functional impact based on high-throughput data from non-coding and intergenic regions of the human genome. It uses experimental data sets from the Encyclopedia of DNA Elements (ENCODE) project [27], public datasets from Gene Expression Omnibus (GEO) [28], and curated published literature.

## Results

### Asthma loci

**Fig. 1:**
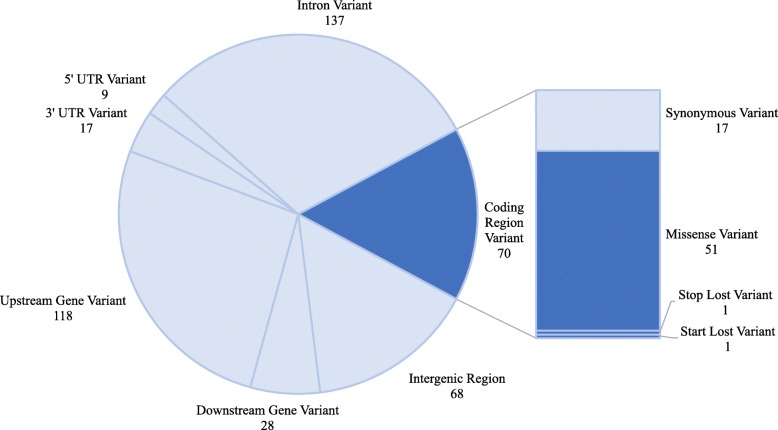
Number of asthma-associated variants by annotation. Genomic variants were previously reported by genome-wide association or candidate gene studies of asthma and asthma related traits. Annotations derived from SnpEff indicate that approximately 12% of the known asthma variants code for a missense or non-sense variant, whereas the majority are non-coding or have no known function

We identified a total of 449 asthma-associated SNPs from earlier GWAS and candidate gene studies. Of these, 225 SNPs were found from asthma GWAS via the NHGRI-EBI GWAS Catalog [13] and mapped to 224 unique genes (Additional file 1: Table S1). In addition, we identified loci reported by candidate gene studies, of which 224 SNPs did not overlap with GWAS loci and mapped to 81 unique genes (Additional file 1: Table S2). Functional annotation of the 449 asthma variants revealed that only 16% are found in protein coding regions, and fewer still (12%) are predicted to cause protein coding changes (Additional file 1: Table S3). The majority of associated variants are intronic (30%), intergenic (15%), or upstream/downstream (32%) gene variants (Fig. 1).

### Linkage disequilibrium variants

**Fig. 2:**
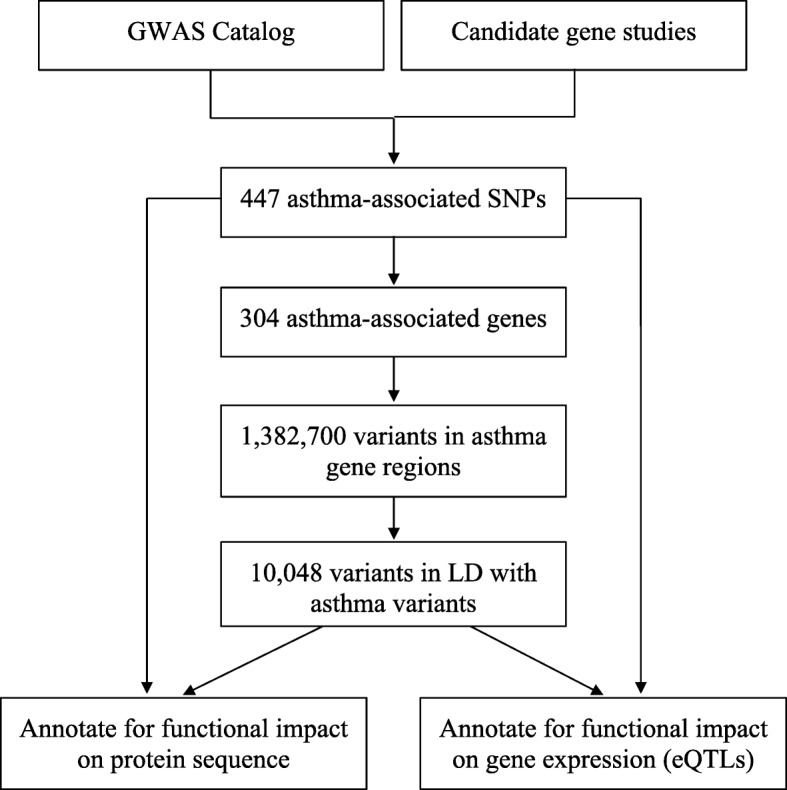
Workflow for discovery of potentially causal variants in known asthma loci. First, we compiled and annotated asthma-associated SNPs from previous GWAS and candidate gene studies of asthma. We then determined the genomic coordinates of the 305 genes and extracted all sequence variations within these loci using data from the 1000 Genomes Project (1000GP). Finally, we identified and annotated those variants in linkage disequilibrium (LD) with known asthma loci to determine which may contribute to changes in protein sequence or regulate gene expression (i.e. expression quantitative trait loci or eQTL)

Using whole-genome sequencing data within the genomic coordinates of the 304 unique asthma genes from GWAS and candidate gene studies, we identified potentially functional variants that are in LD with asthma-associated variants. Specifically, we extracted a total of 1,385,534 variants from these 305 loci using Phase 3 sequence data of the 1000GP. We then assessed LD between the asthma variants and the sequence variants from the 1000GP using data from each of the five continental ancestry groups (African, American, East Asian, European, and South Asian) to account for the discordant nature of LD among ethnic groups [29, 30]. All variants in LD (r^2^ ≥ 0.6) in one or more of the five ancestry groups were included for further analysis. This resulted in 10,130 variants forming 14,908 instances of LD (r^2^ ≥ 0.6) with 345 asthma-associated variants (Additional file 1: Table S4). These 10,130 variants from the 1000GP in LD with one or more asthma variants are hereafter referred to as the ‘LD variants’ and the workflow to generate and annotate them is summarized in Fig. 2. Among the LD variants, 9147 are SNPs, 974 are insertions or deletions, and 9 are structural variants (Additional file 1: Table S5).

### Identification of deleterious and regulatory variants

Many of the LD variants are predicted to have functional consequences on resulting protein structure or to impact regulation of gene expression. Table 1 reports 6 LD variants that are predicted to result in frameshift, stop lost, or splice site mutations by both genomic annotation tools SnpEff and VEP. Two deletions (rs199503730 and rs67841474) are annotated as frameshift variants, both impacting the final coding exon of major histocompatibility complex (MHC) Class I gene *MICA.* The deletion variant rs146576636 in the final coding exon of *ADAM33* is also annotated as a frameshift variant. Two SNPs (rs8084 and rs11078928) are annotated as splice acceptor variants in *HLA-DRA* and *GSDMB* respectively. Finally, the SNP rs15895 is annotated as a stop lost variant in *OAS2*. These variants are in LD with previously reported asthma variants that are less likely to be functional, such as intron variants, upstream/downstream variants, or synonymous variants.

**Table 1:** Deleterious coding variants in LD with asthma-associated SNPs

LD Variant			Asthma Variant			LD r2 value^a^				
ID	Gene	Annotation	ID	Gene(s)	Annotation	AFR	AMR	EAS	EUR	SAS
rs8084	*HLA-DRA*	splice acceptor	rs3129890	*HLA-DRA*	downstream	0.889	< 0.6	0.815	< 0.6	< 0.6
rs199503730	*MICA*	frameshift	rs361525	*TNF, LTA*	upstream	< 0.6	< 0.6	< 0.6	< 0.6	0.726
rs67841474	*MICA*	frameshift	rs2428494	*HLA-B*	upstream	< 0.6	< 0.6	0.671	< 0.6	< 0.6
rs15895	*OAS2*	stop lost	rs1293767	*OAS2*	missense	0.749	0.609	< 0.6	< 0.6	< 0.6
rs11078928	*GSDMB*	splice acceptor	rs907092	*IKZF3*	synonymous	< 0.6	0.889	0.845	0.892	0.966
			rs4795397	*IKZF3*	upstream	< 0.6	0.913	0.995	0.950	0.846
			rs11655198	*ZPBP2*	intron	< 0.6	0.804	0.995	0.832	0.898
			rs2305480	*GSDMB*	missense	0.961	0.988	1	0.996	1
			rs62067034	*GSDMB*	intron	0.707	0.871	1	0.849	0.917
			rs11078927	*GSDMB*	intron	0.984	0.994	1	1	0.995
			rs2290400	*GSDMB*	upstream	< 0.6	0.726	0.945	0.811	0.704
			rs7216389	*GSDMB*	upstream	< 0.6	0.831	0.967	0.792	0.723
			rs4794820	*ORMDL3, LRRC3C*	downstream	< 0.6	0.688	0.899	0.731	0.678
			rs12450323	*IKZF3*	intron	< 0.6	< 0.6	0.684	< 0.6	< 0.6
rs146576636	*ADAM33*	frameshift	rs543749	*ADAM33*	intron	< 0.6	< 0.6	0.804	< 0.6	< 0.6
			rs574174	*ADAM33*	intron	< 0.6	< 0.6	0.846	< 0.6	< 0.6
			rs612709	*ADAM33*	upstream	< 0.6	< 0.6	0.800	< 0.6	< 0.6

*Definition of abbreviations*: *AFR* African population; *AMR* American population; *EAS* East Asian population; *EUR* European population; *LD* Linkage disequilibrium; *SAS* South Asian population

^a^Calculated using 1000 Genomes Project Phase 3 data

In addition to these potentially deleterious coding variants, we identified 91 LD variants that are predicted to be missense variants by both SnpEff and VEP. Of these, 34 are classified as deleterious and/or probably damaging by the pathogenicity prediction tools SIFT and Polyphen-2 (Table 2). The remaining 57 missense LD variants were not identified as deleterious or probably damaging using these software (Additional file 1: Table S6).

**Table 2:** Missense variants in LD with asthma-associated variants

LD Variants			Asthma Variants
ID	Gene	Annotation	ID(s)
rs115676129	*ABI3BP*	missense^a, b^	rs9823506
rs2275254	*CHIA*	missense^a, b^	rs2282290
rs2305479	*GSDMB*	missense^a, b^	rs2305480, rs62067034, rs11078927, rs7216389, rs907092, rs4795397, rs11655198, rs2290400, rs12450323, rs4794820
rs1129808	*HLA-DQA1*	missense^a, b^	rs3104367, rs9272346, rs9273373
rs1142332	*HLA-DQA1*	missense^a, b^	rs3104367, rs9272346, rs9273373
rs1049057	*HLA-DQB1*	missense^a, b^	rs3104367, rs9272346, rs9273373
rs1140319	*HLA-DQB1*	missense^a, b^	rs3104367, rs9272346, rs9273373
rs73022563	*PLEKHG4B*	missense^a, b^	rs62344088
rs6482626	*PTCHD3*	missense^a, b^	rs660498
rs2305089	*TBXT*	missense^a, b^	rs6456042
rs41273547	*ABI3BP*	missense^a^	rs9823506
rs148714608	*CLSTN2*	missense^a^	rs77960860
rs1042308	*HLA-DPA1*	missense^a^	rs987870
rs1071630	*HLA-DQA1*	missense^a^	rs3104367, rs9272346, rs9273373
rs1129740	*HLA-DQA1*	missense^a^	rs3104367, rs9272346, rs9273373
rs1142328	*HLA-DQA1*	missense^a^	rs3104367, rs9272346, rs9273373
rs1142331	*HLA-DQA1*	missense^a^	rs3104367, rs9272346, rs9273373
rs3208105	*HLA-DQA1*	missense^a^	rs3104367, rs9272346, rs9273373
rs1049163	*HLA-DQB1*	missense^a^	rs9273373
rs1130398	*HLA-DQB1*	missense^a^	rs3104367, rs9272346, rs9273373
rs1140318	*HLA-DQB1*	missense^a^	rs3104367, rs9272346, rs9273373
rs1140320	*HLA-DQB1*	missense^a^	rs3104367, rs9272346, rs9273373
rs41558312	*MICA*	missense^a^	rs361525
rs1801280	*NAT2*	missense^a^	rs1799929, rs1208
rs2363468	*PIKFYVE*	missense^a^	rs4673397
rs13436090	*PLEKHG4B*	missense^a^	rs62344088
rs3777134	*SPINK5*	missense^a^	rs2303064
rs682632	*TEK*	missense^a^	rs72721168
rs1140404	*HLA-B*	missense^b^	rs1800629
rs41543121	*HLA-B*	missense^b^	rs1800629
rs41556417	*HLA-B*	missense^b^	rs361525
rs4193	*HLA-DQA1*	missense^b^	rs3104367, rs9272346, rs9273373
rs2233953	*PSORS1C2*	missense^b^	rs1800629
rs7686508	*SEC31A*	missense^b^	rs10022260

*Definition of abbreviations*: *LD* Linkage disequilibrium

^a^Predicted to be deleterious by Sorting Intolerant From Tolerant (SIFT)

^b^Predicted to be probably damaging by Polyphen-2

Finally, we identified LD variants that have been associated with gene expression (i.e. expression quantitative trait loci (eQTL)) using RegulomeDB. We limited our investigation to eQTLs located both at a transcription factor binding site and a DNase peak, two factors characteristic of causal eQTLs [31]. In total, we determined that 24 of the LD variants (Table 3) and 7 of the asthma-associated variants (Additional file 1: Table S7) are known eQTLs.

**Table 3:** Regulatory variants (eQTLs) in LD with asthma-associated variants

eQTL	Affected Gene(s)	Tissue	Affected Binding Motif(s)	Asthma Variant(s) in LD
rs14078	*NPPA*	Lymphoblastoid	C/EBP	rs5065
rs17032120	*UNC50*	Monocytes	Whn	rs2278206
rs3769737	*UNC50*	Monocytes	Gm12892, Hsmm, Hsmmt, Huh7	rs2278206
rs17446058	*UNC50*	Monocytes	deltaEF1	rs2278206
rs5743563	*TLR10, TLR1, TLR6*	Lymphoblastoid	YY1	rs4129009, rs4833095
rs5743565	*TLR10, TLR6, TLR1*	Lymphoblastoid	GATA-4	rs4129009, rs4833095
rs7941648	*RPS6KB2*	Lymphoblastoid	CDP, BCL6, Foxd3, Sox7, Sox18, Sry, Sox5	rs1695, rs6591255
rs12654778	*ADRB2*	Lymphoblastoid	TP53	rs1042713
rs10039559	*RAD50*	Lymphoblastoid	C/EBP	rs11745587
rs204992	*HLA-DQA1*	Lymphoblastoid	CAC-bindingprotein	rs204993
rs3131294	*HLA-DQA1, HLA-C, HLA-DQB1, HLA-DRB1, HLA-DRB5, HLA-DRA, ERG* ^*a*^ *, HCG27, HLA-C, LIMS1* ^*a*^	Lymphoblastoid, Monocytes	AIRE	rs3135388
rs6928482	*HLA-DQA1, HLA-DQA2*	Lymphoblastoid	Zfp410	rs3104367, rs9272346, rs9273373
rs1063355	*HLA-DQA1, HLA-DQB1, HLA-DRB1, HLA-DRA*	Lymphoblastoid	Isgf3g	rs3104367, rs9272346, rs9273373
rs2076523	*HLA-DQA2, HLA-DQB1, HLA-DQA2*	Lymphoblastoid	Hepg2	rs9268516
rs2213585	*HLA-DQB1, HLA-DRB1, HLA-DRB5, HLA-DRA, HLA-DQA1*	Lymphoblastoid	Zfp410	rs3129890
rs6906021	*POLR2A, TAF1, PAX5*	Lymphoblastoid	Pit-1	rs3104367, rs9272346, rs9273373
rs7970524	*HAL, LTA4H*	Monocytes	AR	rs2540493
rs12429692	*ALOX5AP*	Monocytes	PU.1	rs10507391
rs3862469	*DEXI, LOC642755* ^*a*^	Monocytes	NF-E2, ZBRK1, MAFB	rs9923856
rs11642631	*DEXI, LOC642755* ^*a*^	Monocytes	VDR	rs9923856
rs12946510	*KRT222P, MEd24, NR1D1, ORMDL3*	Lymphoblastoid	FOXO1, IRF1, Srf, Elf3	rs907092, rs4795397, rs11655198, rs2305480, rs62067034, rs11078927, rs2290400, rs7216389, rs4794820, rs12450323
rs8076131	*KRT222P, NR1D1, ORMDL3*	Lymphoblastoid	Tcfe2a	rs2305480, rs11078927, rs7216389, rs907092, rs4795397, rs11655198, rs62067034, rs2290400, rs4794820, rs12450323
rs11557466	*ORMDL3*	Lymphoblastoid	HOXA5, REST, NRSE, HIF1	rs2305480, rs62067034, rs11078927, rs907092, rs4795397, rs11655198, rs2290400, rs7216389, rs4794820, rs12450323
rs8069732	*TBKBP1*	Monocytes	NF-E2, Fos, GCN4, hoxa9, Cdx-2, Hoxa11, Hoxc10, Hoxc11, Hoxc9, Hoxa9, Hoxb9	rs5918

*Definition of abbreviations*: *eQTL* expression quantitative trait locus; *LD* linkage disequilibrium

^a^Trans eQTL effect

## Discussion

In this study, we compiled a comprehensive list of asthma-associated variants from earlier candidate gene studies and GWAS in order to conduct a systematic search for causal variants that likely contribute to asthma heritability. Whole genome sequencing data from the 1000GP and functional annotation software tools were used to identify potentially deleterious variants in LD with the asthma-associated variants that could account for the original association signals at these loci. We identified variants annotated to be frameshift, splice site, stop-lost, missense, or eQTLs that are in LD with known asthma variants. The majority of these functional variants were in LD with asthma variants that are non-coding and have no known effects on gene transcription. In summary, we have identified numerous functional variants that could be the elusive causal variants within known asthma-associated loci and improve our understanding of the underlying mechanisms of asthma.

Several of the potentially causal variants identified in this study are found in the MHC class II locus, within the human leukocyte antigen (HLA) super-locus of the 6p21 chromosomal region. MHC class II genes code for proteins that play a central role in the immune system by presenting peptides to CD4+ T cells [32]. The MHC class II locus has been repeatedly associated to asthma and other immune related diseases, but finding causal variants at the locus has proven to be difficult due to its highly polymorphic nature and strong linkage disequilibrium in the region [33, 34]. We compiled 10 asthma risk variants (rs9268516, rs9272346, rs9273349, rs9273373, rs9275698, rs9500927, rs987870, rs3104367, rs3129890, rs7775228) found at this locus from seven GWAS [35–41]. Only two of these asthma-associated variants (rs9272346 and rs7775228) have been reported to regulate gene expression (eQTLs), whereas the remainder are not known to have any functional impact on the resultant transcripts or proteins. The present study identified numerous LD variants within the same loci as these asthma variants including a splice-acceptor variant in the *HLA-DRA* gene (rs8084); missense variants in *HLA-DQA1*, *HLA-DQB1*, *HLA-DRB*, *HLADRB5*, and *HLA-DPA1*; and several eQTLs (rs204992, rs3131294, rs6928482, rs1063355, rs2076523, rs2213585) that are linked to the expression of one or more MHC class II genes. Taken together these LD variants, which are more likely to have functional or even detrimental consequences, could account for the consistent associations between the MHC class II locus and asthma outcomes.

A number of the variants identified in this study are located within the 17q12–21 chromosomal region, which is the most reproducible asthma locus [42, 43]. This genomic region consists of a number of genes, including *ORMDL3*, *GSDMB*, *IKZF3*, *ZPBP2*, and *GSDMA* that are in LD and may either be individually or jointly responsible for the asthma association [42]. The current study compiled 13 asthma-associated variants from the 17q12–21 region (rs3894194, rs4795397, rs62067034, rs7212938, rs7216389, rs11078927, rs2290400, rs2305480, rs4794820, rs6503525, rs11655198, rs12450323, rs907092) that were identified across 11 independent GWAS [37–39, 43–50]. Most of these variants are non-coding, save three missense mutations: rs3894194 and rs7212938 from *GSDMA* and rs2305480 from *GSDMB*. This study identified four additional coding variants from *GSDMA* (rs56030650)*, GSDMB* (rs2305479, rs11078928*)* and *ZPBP2* (rs11557467) and three eQTLs for *ORMDL3* (rs12946510, rs11557466, rs8076131), which are in LD with asthma variants. Notably the LD variant rs11078928 has been functionally characterized as a splice variant that removes an exon, influences transcription levels, and abolishes the biochemical activity of *GSDMB* [51, 52]. This variant has been previously discussed as a possible causal variant in asthma [42, 53] and its LD with multiple asthma-associated variants supports such a hypothesis. The LD variants identified in the 17q12–21 locus demonstrate the possibility that these could represent the causal variants underlying asthma risk.

While we report potentially causal variants in known asthma loci, our study also has some important limitations. For example, given that LD calculations are dependent on allele frequencies and that the majority of known asthma variants are common (minor allele frequency or MAF > 0.05), our study is biased toward identifying LD variants with higher MAF [54]. Additionally, our list of asthma-associated variants from earlier GWAS and candidate gene studies are often identified from a single population and may contain some false positive or population specific results. Further studies are needed to validate these associations in additional asthma populations. Finally, we relied on annotation data from prediction tools such as SnpEff and VEP, which are limited and do not replace experimental evidence. Further studies are needed to identify and ultimately validate the true function, if any, of the LD variants identified in this study to be coding or regulatory. These additional studies include genotyping of these potentially functional variants (identified in LD with the associated variants) in asthma populations and testing them directly for correlation with asthma outcomes. Moreover, functional validation experiments are needed to confirm the biological impact of these variants on the resultant RNA transcripts or proteins, which depends on the predicted impact of the variants identified. For example, variants of high impact (Table 1) including frameshift, splice variants and premature stop codons, could be validated through direct sequencing of transcripts and mass spectrometry to detect truncated and mis-folded proteins.

## Conclusions

The current study is proof of concept that previously correlated loci for asthma may tag causal variants that are in LD, which can be identified using direct sequencing data. While genetic studies of asthma to date have successfully identified hundreds of asthma loci, our understanding of the underlying mechanisms of asthma remains limited. In addition, these loci do not account for all of the asthma heritability and have limited clinical applications due to the fact that the majority of associated variants do not represent the true causative loci. Identification of these causal variants underlying the genetic associations will be instrumental in improving our understanding of the underlying mechanisms of asthma. Ultimately, knowledge of the casual variants will help to facilitate the development of more accurate clinical tests for determining risk and treatment options for asthma.

## Additional file

Additional file 1: **Table S1.** Asthma-associated SNPs from genome-wide association studies. **Table S2.** Asthma-associated SNPs from candidate gene studies. **Table S3.** Asthma-associated variants with protein coding annotation. **Table S4.** Ethnic specific linkage disequilibrium between asthma variants and 1000GP variants. **Table S5.** Annotation of all variants in linkage disequilibrium with asthma variants. **Table S6.** Missense variants not predicted to be deleterious in linkage disequilibrium with asthma-associated variants. **Table S7.** eQTLs within asthma-associated variants. (XLSX 1149 kb)

## Abbreviations

1000GP: 1000 Genomes Project
eQTL: Expression quantitative trait loci
GWAS: Genome-wide association study
HLA: Human leukocyte antigen
LD: Linkage disequilibrium
MHC: Major histocompatibility complex
NHGRI-EBI: National Human Genome Research Institute-European Bioinformatics Institute
SIFT: Sorting Intolerant From Tolerant
SNP: Single nucleotide polymorphism
VEP: Variant Effect Predictor
